# Heat-Inactivated *Lactiplantibacillus plantarum* FRT4 Alleviates Diet-Induced Obesity via Gut–Liver Axis Reprogramming

**DOI:** 10.3390/foods14162799

**Published:** 2025-08-12

**Authors:** Yuyin Huang, Qingya Wang, Xiling Han, Kun Meng, Guohua Liu, Haiou Zhang, Rui Zhang, Hongying Cai, Peilong Yang

**Affiliations:** 1 Key Laboratory of Feed Biotechnology of Ministry of Agriculture and Rural Affairs, Institute of Feed Research, Chinese Academy of Agricultural Sciences, Beijing 100081, China; yuyinhuang99@163.com (Y.H.); mengkun@caas.cn (K.M.); liuguohua@caas.cn (G.L.); zhanghaiou@caas.cn (H.Z.); 2 Key Laboratory of Yunnan for Biomass Energy and Biotechnology of Environment, Yunnan Normal University, Kunming 650500, China; 18238693320@163.com (Q.W.); 17789363681@163.com (X.H.); ruizhang@ynnu.edu.cn (R.Z.)

**Keywords:** *Lactiplantibacillus plantarum* FRT4, heat-inactivated probiotics, obesity, gut–liver axis, short-chain fatty acids, lipid metabolism

## Abstract

Obesity and related metabolic disorders are major global health challenges. Postbiotics, such as heat-inactivated probiotics, have attracted attention for their improved safety, stability, and potential metabolic benefits compared to live probiotics. However, the comparative anti-obesity effects and mechanisms of live versus heat-inactivated *Lactiplantibacillus plantarum* FRT4 remain unclear, so this study systematically evaluated their effects and mechanisms in high-fat-diet-induced obese mice. Mice received oral administration of live or heat-inactivated FRT4 (prepared by heating in a water bath at 80 °C for 5 min) for 16 weeks. Comprehensive analyses included metabolic profiling, histological evaluation, serum and liver biomarkers, gut microbiota composition, liver metabolomics, and transcriptomics. Both live and inactivated FRT4 significantly reduced body weight gain, adiposity, hepatic steatosis, and dyslipidemia, with inactivated FRT4 exhibiting comparable or superior efficacy. Notably, inactivated FRT4 restored gut microbiota composition, increased short-chain fatty acid production, and regulated hepatic metabolic pathways. Multi-omics analyses revealed modulation of lipid biosynthesis, amino acid metabolism, and energy utilization pathways. Specifically, the “biosynthesis of unsaturated fatty acids” pathway was downregulated in metabolomics and significantly enriched in transcriptomics, highlighting its central role in FRT4M-mediated metabolic reprogramming. These findings demonstrate that heat-inactivated *Lp. plantarum* FRT4 exerts systemic anti-obesity effects via gut–liver axis modulation, supporting its potential as a promising postbiotic intervention for obesity and metabolic dysfunction.

## 1. Introduction

Obesity is characterized by excessive accumulation of adipose tissue and is closely associated with a range of metabolic disturbances, such as insulin resistance, dyslipidemia, and chronic low-grade inflammation [1,2,3]. In recent years, the global prevalence of obesity has increased substantially, primarily driven by the adoption of sedentary lifestyles and greater consumption of energy-dense, high-fat diets [4]. According to the latest report from the World Health Organization (WHO), more than 1 billion people worldwide were living with obesity in 2022, including 890 million adults, and 160 million young people (adolescents and children). The global prevalence of obesity has nearly tripled since 1975, and in 2022, 43% of adults were considered overweight, with 890 million classified as obese [5]. These alarming statistics underscore the urgent need for effective strategies to prevent and manage obesity and its related complications. Although pharmacological interventions and bariatric surgery are available for obesity treatment, their long-term application is often limited by high costs, poor patient compliance, and potential adverse effects [6,7]. Therefore, it is imperative to develop safer, more stable, and sustainable approaches for obesity intervention.

In recent years, accumulating evidence has identified the gut and liver as critical regulators in the onset and progression of obesity [8,9]. The gut serves not only as the primary site for nutrient absorption but also plays a pivotal role in host metabolism and immune modulation through its complex microbial ecosystem [10]. The gut microbiota influences lipid metabolism by producing short-chain fatty acids (SCFAs), regulating bile acid homeostasis, and maintaining intestinal barrier integrity [11,12,13]. A high-fat diet often disrupts the microbial balance, leading to a reduction in beneficial bacteria, decreased SCFA production, and increased intestinal permeability [14,15,16]. Gut-derived metabolites, microbial components, and inflammatory signals can translocate to the liver via the portal vein, triggering inflammatory pathways that promote hepatic lipid accumulation, insulin resistance, and metabolic dysregulation [17,18,19]. Conversely, intestinal high-density lipoprotein can also reach the liver through the portal circulation and mitigate hepatic injury [20,21]. Meanwhile, as the central organ for lipid synthesis and metabolism, the liver is closely tied to systemic energy homeostasis [22]. Therefore, this bidirectional communication, known as the gut–liver axis, is increasingly recognized as a key factor in the development of obesity and related metabolic disorders.

Given the central role of the gut–liver axis in metabolic homeostasis, targeted modulation of this pathway represents a promising strategy for obesity intervention. Among various approaches, probiotics, particularly *Lactiplantibacillus plantarum* strains, can beneficially modulate gut microbiota composition and reinforce barrier function, thereby influencing the gut–liver axis and improving metabolic health [23,24,25]. Our previous studies demonstrated that *Lp. plantarum* FRT4 effectively alleviates body weight gain, hepatic lipid accumulation, and dyslipidemia in obese animal models [26]. However, the viability of live probiotics can decline during processing, storage, and gastrointestinal transit, which limits their broader application in functional foods and clinical interventions [27]. Against this backdrop, inactivated probiotics, also known as parabiotics, have emerged as a promising nutritional strategy due to their enhanced stability and safety profiles [28,29]. Different from traditional probiotics, inactivated strains lack viability but may still exert biological effects through their cell wall components, intracellular substances, or metabolic by-products by modulating immune and metabolic pathways. Preliminary evidence has shown that heat-inactivated *Lp. plantarum* possesses potential anti-inflammatory, antioxidant, and metabolic regulatory properties [30,31]. However, the precise mechanisms underlying its anti-obesity effects remain unclear, particularly with respect to its role in regulating the gut–liver axis.

In this study, both live and heat-inactivated *Lp. plantarum* FRT4 were investigated in a high-fat-diet (HFD)-induced obese mouse model to determine whether both forms could alleviate obesity-related metabolic disturbances, and to compare the magnitude of their effects on body weight management, lipid regulation, and liver protection. By integrating 16S rRNA sequencing of the gut microbiota, untargeted hepatic metabolomics, and transcriptomic profiling, we systematically explored the anti-obesity mechanisms of FRT4 from multiple perspectives, including gut microbiota, metabolic pathways, and gene expression, with particular emphasis on regulatory effects involving the gut–liver axis. This study aims to provide theoretical support and experimental evidence for the application of both live and inactivated probiotics as functional food components for the intervention of metabolic disorders.

## 2. Materials and Methods

### 2.1. Preparation of Live and Heat-Inactivated Lp. plantarum FRT4

As in our previous study [32], *Lp. plantarum* FRT4 was activated by culturing in MRS broth at 37 °C for 24 h. After two consecutive activations, the bacterial culture was centrifuged at 4000 rpm for 10 min at 4 °C to remove the supernatant. The cell pellet was washed with sterile 0.9% (*w*/*v*) saline and resuspended in saline to a final concentration of 1 × 10^10^ CFU/mL. To prepare heat-inactivated *Lp. plantarum* FRT4, the bacterial suspension was subjected to heat treatment in a water bath at 80 °C for 5 min. After heat treatment, the suspension was immediately plated onto MRS agar and incubated at 37 °C for 48 h. The absence of colony formation confirmed that all FRT4 cells were completely inactivated.

### 2.2. Animals Experiment

A total of 40 male C57BL/6J mice (6 weeks old, body weight 20 ± 1 g) were purchased from Beijing Vital River Laboratory Animal Technology Co., Ltd. (Beijing, China). The mice were housed under controlled conditions (22 ± 1 °C, 55 ± 10% humidity) with a 12 h light/dark cycle. All animal procedures were conducted in strict accordance with the Guide for the Care and Use of Laboratory Animals issued by the National Research Council. The experimental protocols were approved by the Animal Ethics Committee of the Institute of Feed Research, Chinese Academy of Agricultural Sciences (CAAS) under license number IFR-CAAS20240415. Before the experiment, all mice were allowed free access to standard chow and water during a 7-day acclimation period. After acclimation, mice were randomly divided into five groups (*n* = 8 per group): control group (CT), high-fat-diet model group (HFD), positive control group (PC; atorvastatin calcium tablets, 30 mg/kg), live *Lp. plantarum* FRT4 group (FRT4H; 2 × 10^9^ CFU/day), and heat-inactivated *Lp. plantarum* FRT4 group (FRT4M; 2 × 10^9^ CFU/day). The dosage of 2 × 10^9^ CFU/day was selected based on our previous studies and the published literature [26,33,34,35,36]. The dosage of 30 mg/kg for atorvastatin was selected according to our previous studies [34,37]. The CT group received a standard SPF diet, while the other groups were fed a high-fat diet (60% kcal from fat, 20% protein, and 20% carbohydrate; D12492-type, Beijing Keao Xieli Feed Co., Ltd., Beijing, China). All mice were administered substances once daily via oral gavage throughout the intervention. Mice in the CT and HFD groups received 200 μL of sterile saline daily, while the treatment groups received the corresponding reagents. Body weight and food intake were recorded weekly throughout the 16-week experimental period.

### 2.3. Sample Collection

At the end of the experiment, all mice were fasted for 16 h. Following anesthesia, blood samples were collected via retro-orbital bleeding and allowed to clot at room temperature for 2 h. The blood was centrifuged at 3000× *g* for 10 min at 4 °C, and the resulting serum was rapidly frozen in liquid nitrogen and subsequently stored at −80 °C for further analysis. After blood collection, all 40 mice were euthanized. The liver, white adipose tissues (WATs), spleen, and kidneys were carefully excised, weighed immediately, and recorded. Organ indices were calculated based on the organ-to-body weight ratio to assess systemic organ hypertrophy caused by HFD and its potential improvement following FRT4 or atorvastatin intervention. Portions of liver and WATs were fixed in 4% neutral buffered paraformaldehyde for histological analysis, while the remaining tissue samples were stored at −80 °C for subsequent biochemical and molecular analyses.

### 2.4. Biochemical Assay of Serum and Liver Tissues

Serum levels of total cholesterol (TC), triglycerides (TG), high-density lipoprotein cholesterol (HDL-C), low-density lipoprotein cholesterol (LDL-C), alanine aminotransferase (ALT), aspartate aminotransferase (AST), lactate dehydrogenase (LDH), glucose (GLU), and non-esterified fatty acids (NEFAs) were measured using commercial assay kits. In addition, hepatic levels of ALT, AST, GLU, HDL-C, LDL-C, TC, TG, and very low-density lipoprotein cholesterol (VLDL-C) were also determined. All biochemical kits were purchased from Shanghai Enzyme-linked Biotechnology Co., Ltd. (Shanghai, China), and all procedures were strictly performed in accordance with the manufacturer’s instructions provided in the kit manuals. The inter-assay coefficients of variation (CV) for all kits were below 5%, and the sensitivity for each assay was within the predefined detection range stated by the manufacturer.

### 2.5. Oil Red O Staining of Liver Tissue

Liver tissues were fixed in 4% buffered paraformaldehyde solution for 24 h. Fixed liver tissues were dehydrated through a graded ethanol series and embedded in optimal cutting temperature compound for cryopreservation. Frozen sections with a thickness of 4–5 μm were prepared using a cryostat. Oil Red O (ORO) staining, specific for neutral lipids, was performed under light-protected conditions to prevent dye degradation. The staining procedure included differentiation in 60% isopropanol, followed by counterstaining with hematoxylin to visualize nuclei. After staining, sections were mounted with glycerol gelatin mounting medium to preserve the stained lipids. Stained sections were observed under a NIKON ECLIPSE E100 light microscope (Nikon Corporation, Tokyo, Japan) equipped with a DS-U3 digital imaging system (Nikon, Japan) for histological and lipid accumulation analysis.

### 2.6. Hematoxylin and Eosin (H&E) Staining of Liver and WATs

Fixed liver and WATs were dehydrated through a graded ethanol series and cleared in xylene. The cleared tissues were embedded in molten paraffin and allowed to solidify in embedding molds. Paraffin blocks were trimmed and cooled on a −20 °C cryo-plate before sectioning. Tissue sections (4–5 μm thick) were cut using a rotary microtome and mounted on glass slides. After deparaffinization and rehydration, sections were stained with hematoxylin followed by eosin. Stained sections were dehydrated, coverslipped, and examined under a light microscope. Images were captured and analyzed using standard imaging software. Histological evaluation was performed qualitatively by independent pathologists blinded to group allocation.

### 2.7. 16S rRNA Gene Sequencing and Microbiota Analysis

To investigate the effects of lactic acid bacteria on gut microbiota in obese mice, a total of 30 cecal content samples were collected and subjected to 16S rRNA gene amplicon sequencing on the Illumina NovaSeq 6000 platform (Biomarker Technologies, Beijing, China). Microbial DNA was extracted using the TGuide S96 Magnetic Stool DNA Kit following the manufacturer’s instructions. The V3–V4 hypervariable regions of the 16S rRNA gene were amplified using universal primers 338F and 806R. Amplicons were purified with Agencourt AMPure XP beads, quantified using the Qubit dsDNA HS Assay Kit and Qubit 4.0 fluorometer, and pooled in equimolar concentrations for library construction. Sequencing was performed on the NovaSeq platform. Sequence denoising, paired-end merging, and chimera removal were performed using the DADA2 pipeline in QIIME2 (version 2020.6) to generate amplicon sequence variants (ASVs), as previously described [38,39].

### 2.8. Analysis of SCFAs in Cecal Contents

To investigate the effects of lactic acid bacteria on SCFA production, approximately 2 g of cecal contents was transferred into 2 mL Eppendorf tubes and mixed with 1 mL of distilled water. After vortexing for 10 s, the samples were homogenized using an automatic sample grinder at 40 Hz for 4 min. The homogenates were subjected to ultrasonic extraction in an ice-water bath for 5 min, and this cycle was repeated three times. The mixture was then centrifuged at 5000× *g* for 20 min at 4 °C. A total of 0.8 mL of the supernatant was transferred into a new 2 mL Eppendorf tube, followed by the addition of 0.1 mL of 50% sulfuric acid and 0.8 mL of methyl tert-butyl ether containing 25 mg/L of 2-methylvaleric acid as an internal standard. After vortexing for 10 s and shaking for 10 min, the samples were further sonicated in an ice bath for 10 min. Subsequently, samples were centrifuged at 10,000× *g* for 15 min at 4 °C. The resulting supernatants were stored at −20 °C for 30 min before being transferred into 2 mL glass vials for further gas chromatography–mass spectrometry (GC–MS) analysis. SCFAs were analyzed using a Shimadzu GC2030-QP2020 NX gas chromatography–mass spectrometry system equipped with a high-polarity capillary column (HP-FFAP). The injection volume was 1 μL in split mode (5:1 split ratio), and helium was used as the carrier gas. The purge flow was set at 3 mL/min, and the column flow rate was 1.2 mL/min. The temperature program was as follows: initial temperature 50 °C for 1 min; ramped at 50 °C/min to 150 °C (hold 1 min); then 10 °C/min to 170 °C; 25 °C/min to 225 °C (hold 1 min); and finally, 40 °C/min to 240 °C (hold 1 min). The inlet, transfer line, quadrupole, and ion source temperatures were set at 220 °C, 240 °C, 150 °C, and 240 °C, respectively. The MS was operated in electron impact (EI) mode at −70 eV. Data were acquired under full scan/SIM mode with a solvent delay of 3.75 min and a mass scan range of *m*/*z* 33–150.

### 2.9. Untargeted Metabolomics Analysis of Liver Tissue

Approximately 25 mg of tissue was placed in an Eppendorf tube and extracted with 500 μL of methanol–acetonitrile–water (2:2:1, *v*/*v*/*v*) containing isotopically labeled internal standards. After vortexing, samples were homogenized (35 Hz, 4 min) and sonicated in an ice-water bath for 5 min; this cycle was repeated three times. Samples were then incubated at −40 °C for 1 h and centrifuged at 12,000 rpm for 15 min at 4 °C. A 100 μL aliquot of the supernatant was transferred to autosampler vials for LC–MS/MS analysis. Quality control (QC) samples were prepared by pooling equal volumes of supernatants from all samples. Polar metabolites were separated using a Vanquish UHPLC system (Vanquish, Thermo Fisher Scientific, Waltham, MA, USA) equipped with a Waters ACQUITY UPLC BEH Amide column (2.1 mm × 50 mm, 1.7 μm; Waters Corporation, Milford, MA, USA). The mobile phases consisted of 25 mM ammonium acetate with 25 mM ammonia in water and acetonitrile. The column temperature was maintained at 4 °C, and the injection volume was 2 μL. Mass spectrometry was performed on an Orbitrap Exploris 120 (Orbitrap MS, Thermo Fisher Scientific, Waltham, MA, USA) operated in both MS1 and MS2 modes. Raw data were converted to mzXML format and analyzed in R using the BiotreeDB (v3.0) database for metabolite identification and visualization. Metabolite annotation confidence levels were assigned based on the guidelines of the Metabolomics Standards Initiative (MSI). Specifically, metabolites matched with MS1 *m*/*z*, retention time (RT), and MS/MS spectra from authentic standards were classified as Level 1. Metabolites matched by MS1 and MS/MS without RT were assigned as Level 2. This classification is consistent with the KGMN framework implemented in MetDNA2 [40]. To control false positives in downstream analyses, *p*-values were adjusted using the false discovery rate (FDR) method, with a significance threshold set at 5% (FDR < 0.05). This threshold was used for both differential metabolite analysis and pathway enrichment.

### 2.10. Liver Transcriptome Sequencing and Analysis

Total RNA was extracted from liver tissue and assessed for purity, concentration, and integrity using NanoDrop 2000 and the Agilent 2100 Bioanalyzer. High-quality RNA samples were used to construct cDNA libraries by enriching mRNA with oligo(dT) magnetic beads, followed by fragmentation, cDNA synthesis, end repair, A-tailing, adaptor ligation, and PCR amplification. Libraries were quantified using Qubit 3.0 and QPCR, and fragment sizes were validated with the Qsep400 system. Sequencing was performed on an Illumina platform using paired-end 150 bp (PE150) reads. Each library generated between 38.5 and 47.6 million clean reads, and the mapping efficiency to the mouse reference genome ranged from 95.42% to 97.56%, indicating high sequencing quality and alignment reliability. Raw data were processed on the BMKCloud platform (www.biocloud.net), including quality control, genome alignment, transcript assembly, differential expression analysis, and functional annotation.

### 2.11. Statistical Analysis

All data are expressed as mean ± standard deviation (SD). One-way analysis of variance (ANOVA), followed by multiple comparisons using Dunnett’s post hoc test, was used to compare treatment groups against the HFD group. For pairwise comparisons between groups, Student’s *t*-test was applied. Effect sizes were calculated using Cohen’s d for *t*-tests and eta-squared (η^2^) for ANOVA. A *p*-value less than 0.05 was considered statistically significant. Graphical representations and statistical analyses were performed using GraphPad Prism 8 (GraphPad Software, San Diego, CA, USA). For metabolomic and transcriptomic data, *p*-values were adjusted using the false discovery rate (FDR) method, and FDR-adjusted *p*-values < 0.05 were considered statistically significant. Although a formal a priori power analysis was not conducted, the sample sizes were determined based on previously published studies with similar designs, effect sizes, and variability [34,37].

## 3. Results

### 3.1. Inactivated Lp. plantarum FRT4 Alleviates HFD-Induced Body Weight Gain and Organ Hypertrophy in Mice

**Figure 1:**
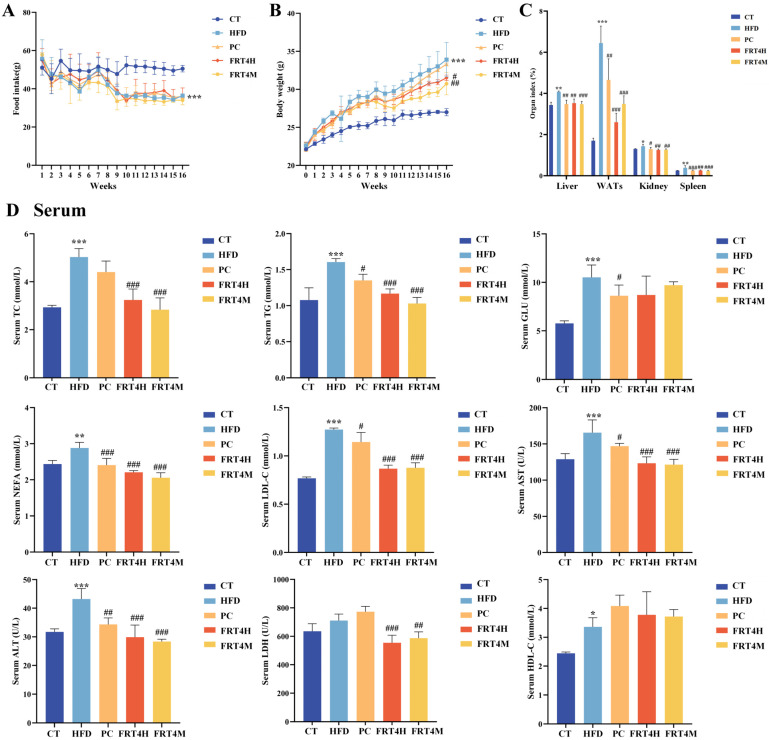
Inactivated *Lp. plantarum* FRT4 alleviates obesity and serum lipid accumulation in HFD-fed mice. (**A**) Food intake. (**B**) Body weight gain over the feeding period. (**C**) Organ indices of liver, WATs, kidney, and spleen. (**D**) Serum biochemical parameters. *N* = 8 mice per group. The symbols *, **, and *** indicate significant differences at the *p* < 0.05, *p* < 0.01, and *p* < 0.001 levels, respectively, when comparing the HFD and CT groups. Similarly, the symbols #, ##, and ### denote significant differences at *p* < 0.05, *p* < 0.01, and *p* < 0.001 for the comparison between the PC, FRT4H, or FRT4M and HFD groups.

During the HFD feeding period, the food intake of mice in the HFD group was significantly reduced compared to the CT group (*p* < 0.001). However, there were no significant differences in food intake among the HFD, FRT4H, FRT4M, and PC groups (Figure 1A, *p* > 0.05), indicating that FRT4M intervention did not affect feeding behavior. As shown in Figure 1B, HFD significantly accelerated body weight gain in mice (*p* < 0.001). Interventions with FRT4H, FRT4M, and PC effectively mitigated this trend. By week 16, the body weight of mice in the HFD group was significantly higher than that of the CT group (*p* < 0.001), while FRT4H (*p* < 0.05) and FRT4M (*p* < 0.01) supplementation significantly suppressed this weight gain. Notably, the inactivated FRT4 exhibited superior effects compared to the live form. As presented in Figure 1C, organ indices (liver, WATs, kidney, and spleen) were significantly increased in the HFD group compared to the CT group (*p* < 0.05), consistent with the final body weight trends. This indicates that HFD not only induces weight gain but also leads to organ hypertrophy. However, following interventions with FRT4H, FRT4M, and atorvastatin, these organ indices were significantly reduced (*p* < 0.05). These findings suggest that inactivated *Lp. plantarum* FRT4, similar to its live counterpart, effectively alleviates HFD-induced body weight gain and organ enlargement in obese mice.

### 3.2. Inactivated Lp. plantarum FRT4 Improves Serum and Hepatic Biochemical Parameters

As shown in Figure 1D, compared with the CT group, the HFD group exhibited significantly elevated levels of serum TC, TG, GLU, LDL-C, AST, ALT (*p* < 0.001), and NEFA (*p* < 0.01). Both live and inactivated FRT4 effectively alleviated the increases of TC, TG, NEFA, LDL-C, AST, and ALT (*p* < 0.001). No significant difference in LDH levels was observed between the CT and HFD groups; however, LDH levels were significantly reduced in the FRT4H, FRT4M, and PC groups compared to the HFD group. Additionally, there were no significant differences in HDL-C levels among the HFD, PC, FRT4H, and FRT4M groups.

**Figure 2:**
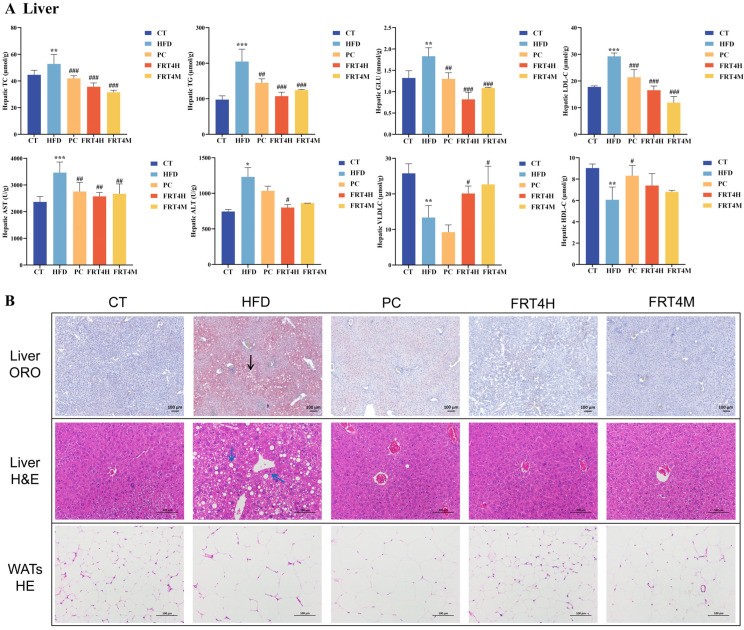
Inactivated *Lp. plantarum* FRT4 alleviates hepatic lipid accumulation and histopathological alterations in the liver and adipose tissue of HFD-fed mice. (**A**) Hepatic biochemical parameters. *N* = 8 mice per group. The symbols *, **, and *** indicate significant differences at the *p* < 0.05, *p* < 0.01, and *p* < 0.001 levels, respectively, when comparing the HFD and CT groups. Similarly, the symbols #, ##, and ### denote significant differences at *p* < 0.05, *p* < 0.01, and *p* < 0.001 for the comparison between the PC, FRT4H, or FRT4M and HFD groups. (**B**) Histological images: ORO staining of liver tissue (10×), H&E staining of liver and WATs (200×). The accumulation of lipid droplets is indicated by the increased intensity and area of red staining in the ORO images. The black arrow indicate lipid vacuoles formed by partial dissolution or displacement of fat droplets during tissue processing. In the HFD group of HE staining of liver, blue arrows indicate intracellular vacuoles and damaged hepatocytes. Scale bar = 100 μm.

In the liver (Figure 2A), the levels of TC, TG, GLU, LDL-C, AST, and ALT were significantly increased in HFD-fed mice compared to the CT group (*p* < 0.01). Both live and inactivated FRT4 effectively mitigated these elevations. Furthermore, HFD significantly reduced hepatic HDL-C and VLDL-C levels (*p* < 0.01), while FRT4 interventions notably increased VLDL-C levels. Although HDL-C levels were also elevated by FRT4 treatment, the changes were not statistically significant.

### 3.3. Inactivated Lp. plantarum FRT4 Reduces Hepatic Lipid Accumulation and Attenuates Hepatic and WAT Lesions

As shown in Figure 2B, ORO staining of liver sections showed extensive lipid droplet accumulation in the HFD group, suggesting significant hepatic lipid deposition and tissue degeneration induced by the high-fat diet. In contrast, hepatic lipid accumulation was markedly reduced in mice treated with live FRT4, inactivated FRT4, or PC, indicating the protective effects of FRT4 against diet-induced hepatic steatosis.

H&E staining of liver tissue revealed that hepatocytes in the CT group exhibited intact structures, clear cellular boundaries, and no signs of steatosis. In contrast, the HFD group exhibited prominent intracellular vacuoles, which were clear, round, or oval spaces in the cytoplasm of hepatocytes, and they correspond to lipid droplets dissolved during tissue processing. In addition, numerous hepatocytes in this group displayed signs of injury, including cell swelling, cytoplasmic rarefaction, and loss of cellular boundaries. Following treatment with live FRT4, inactivated FRT4, or PC, the number and size of damaged hepatocytes were significantly reduced, with improved cellular morphology and fewer vacuoles, closely resembling the normal hepatic structure.

H&E staining of WATs demonstrated that adipocytes in the HFD group were markedly hypertrophic with irregular morphology compared to those in the CT group. Intervention with live FRT4, inactivated FRT4, or atorvastatin resulted in visibly smaller and more uniformly shaped adipocytes, with clearer cell boundaries and reduced cytoplasmic distortion. These improvements were qualitatively assessed by independent pathologists in a blinded manner, indicating a reversal of HFD-induced adipocyte degeneration and cellular injury at the histological level.

### 3.4. Inactivated Lp. plantarum FRT4 Improves Gut Microbiota Dysbiosis

**Figure 3:**
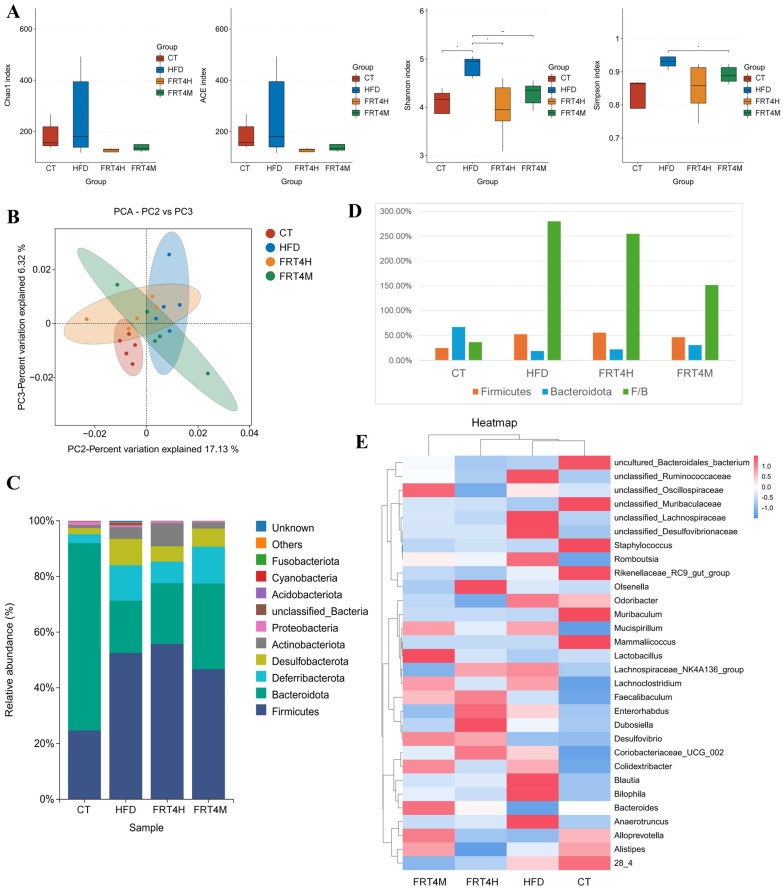
Inactivated *Lp. plantarum* FRT4 modulates gut microbiota composition in HFD-fed mice. (**A**) Alpha diversity indices (Chao1, ACE, Simpson, and Shannon). (**B**) PCA plot of gut microbiota composition. The symbols *, ** indicate significant differences at the *p* < 0.05, *p* < 0.01 levels. (**C**) Relative abundance of gut microbiota at the phylum level. (**D**) Firmicutes/Bacteroidetes (F/B) ratio. (**E**) Heatmap of microbial composition at the genus level. *N* = 5 mice per group.

The effects of inactivated FRT4 on gut microbiota composition in HFD-fed mice were investigated using 16S rRNA sequencing. As shown in Figure 3A, compared to the CT group, the HFD group exhibited increased Chao1, ACE, Simpson, and Shannon indices, indicating that HFD significantly altered the richness and diversity of the gut microbiota. Following intervention with live or inactivated FRT4, these indices were reduced, suggesting a shift in the microbial community structure toward that of the CT group. Principal component analysis (PCA) revealed a clear separation between the CT and HFD groups (Figure 3B), highlighting significant differences in overall microbial composition induced by the high-fat diet. Notably, the microbial profiles of the FRT4H and FRT4M groups were distinctly separated from those of the HFD group and were more closely aligned with the CT group. These results suggest that both live and inactivated FRT4 effectively alleviate HFD-induced gut microbiota dysbiosis and partially restore the microbial community structure toward a healthier profile.

The composition of the gut microbiota at the phylum level is shown in Figure 3C. A common feature of gut microbiota in obese humans and mice is an increased Firmicutes-to-Bacteroidota (F/B) ratio. In the present study, compared with the CT group, the HFD group exhibited a higher relative abundance of Firmicutes and a decreased abundance of Bacteroidota, resulting in a significantly elevated F/B ratio (Figure 3D). Intervention with either live or inactivated FRT4 significantly reduced the F/B ratio, with inactivated FRT4 showing the most pronounced effect.

At the genus level (Figure 3E), the HFD group showed a marked decrease in the relative abundance of *uncultured_Bacteroidales_bacterium*, *unclassified_Muribaculaceae*, *Muribaculum*, *Lactobacillus*, *Bacteroides*, *Alloprevotella*, *Alistipes*, and *Lachnospiraceae bacterium 28_4* compared to the CT group. In contrast, genera such as *unclassified_Lachnospiraceae*, *unclassified_Desulfovibrionaceae*, *Romboutsia*, *Odoribacter*, *Mucispirillum*, *Lachnospiraceae_NK4A136_group*, *Lachnoclostridium*, *Dubosiella*, *Coriobacteriaceae_UCG_002*, *Colidextribacter*, *Blautia*, *Bilophila*, and *Anaerotruncus* were significantly increased in the HFD group. Both live and inactivated FRT4 reversed these alterations to varying degrees. Specifically, in the inactivated FRT4 group, the abundances of *uncultured_Bacteroidales_bacterium*, *unclassified_Muribaculaceae*, *Lactobacillus*, *Bacteroides*, *Alloprevotella*, and *Alistipes* were increased, while those of *unclassified_Lachnospiraceae*, *unclassified_Desulfovibrionaceae*, *Romboutsia*, *Odoribacter*, *Lachnospiraceae_NK4A136_group*, *Dubosiella*, *Coriobacteriaceae_UCG_002*, *Blautia*, and *Bilophila*, and *Anaerotruncus* were decreased compared to the HFD group. In the live FRT4 group, *unclassified_Muribaculaceae*, *Lactobacillus*, *Bacteroides*, and *Alloprevotella* were significantly increased, whereas *unclassified_Lachnospiraceae*, *unclassified_Desulfovibrionaceae*, *Romboutsia*, *Odoribacter*, *Mucispirillum*, *Lachnoclostridium*, *Colidextribacter*, *Blautia*, *Bilophila*, and *Anaerotruncus* were decreased.

### 3.5. Inactivated Lp. plantarum FRT4 Enhances Intestinal SCFA Levels

**Figure 4:**
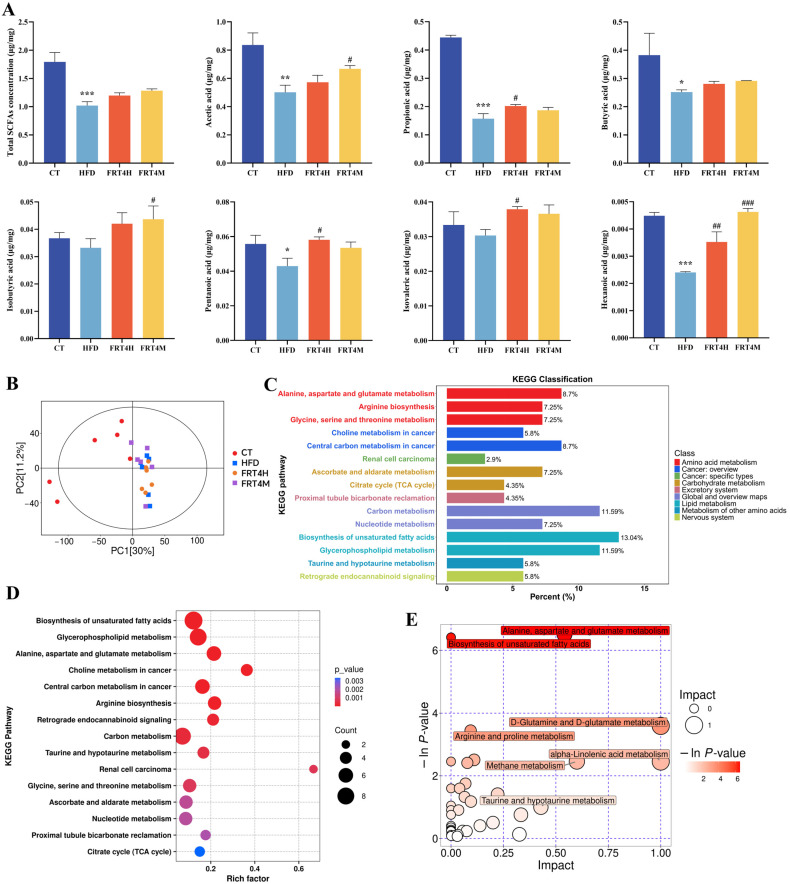
Inactivated *Lp. plantarum* FRT4 increases SCFA levels and alleviates hepatic metabolic disturbances in HFD-fed mice. (**A**) SCFA levels in cecal contents. *n* = 5 mice per group. (**B**) PCA plot of liver metabolomics. The symbols *, **, and *** indicate significant differences at the *p* < 0.05, *p* < 0.01, and *p* < 0.001 levels, respectively, when comparing the HFD and CT groups. Similarly, the symbols #, ##, and ### denote significant differences at *p* < 0.05, *p* < 0.01, and *p* < 0.001 for the comparison between the PC, FRT4H, or FRT4M and HFD groups. (**C**) KEGG pathway classification of differential metabolites. (**D**) KEGG pathway enrichment bubble plot. The x-axis shows the rich factor, and the y-axis lists pathway names. Bubble size indicates the number of matched metabolites, and color represents the adjusted *p*-value. Darker red color corresponds to more significant enrichment (smaller *p*-values). (**E**) Topological enrichment bubble plot. Each bubble represents a pathway. The x-axis indicates the pathway impact value calculated by topological analysis. The y-axis shows the statistical significance as –ln(*p*-value). Bubble size reflects impact value, and bubble color intensity indicates enrichment significance. Pathways with both high impact and significance are labeled. *N* = 6 mice per group.

SCFAs in the host gut are closely associated with the composition of the intestinal microbiota. As shown in Figure 4A, compared with the CT group, the HFD group exhibited significantly reduced levels of total SCFAs, including acetic acid, propionic acid, butyric acid, pentanoic acid, and hexanoic acid (*p* < 0.05). Compared with the HFD group, mice treated with inactivated FRT4 showed significant increases in acetic acid, isobutyric acid, and hexanoic acid (*p* < 0.05). And total SCFAs, propionic acid, butyric acid, pentanoic acid, and isovaleric acid were also elevated without significant differences statistically. In the live FRT4 group, the levels of propionic acid, pentanoic acid, isovaleric acid, and hexanoic acid were significantly increased compared to the HFD group (*p* < 0.05). Overall, inactivated FRT4 demonstrated a more pronounced effect than the live strain in promoting SCFA production in HFD-fed mice, especially in total SCFAs.

### 3.6. Inactivated Lp. plantarum FRT4 Alleviates Hepatic Metabolic Disturbances

The liver is a central organ for lipid metabolism. To investigate the metabolic regulatory effects of FRT4, non-targeted metabolomic profiling was performed on liver tissues from CT, HFD, live FRT4, and inactivated FRT4 groups. As shown in the PCA plot (Figure 4B), the HFD group was distinctly separated from the CT group, indicating significant alterations in hepatic metabolism due to a high-fat diet. Notably, both FRT4H and FRT4M groups also showed separation from the HFD group, with FRT4M exhibiting a more pronounced shift, suggesting a stronger regulatory effect. KEGG classification of differential metabolites among the four groups (Figure 4C) revealed that the most enriched category was lipid metabolism, including “Biosynthesis of unsaturated fatty acids” (13.04%) and “Glycerophospholipid metabolism” (11.59%), indicating that lipid metabolism was significantly affected by both HFD and probiotic intervention. Additionally, carbon metabolism accounted for 11.59%, highlighting its close relationship with lipid biosynthesis. Amino acid metabolism was also highly represented in the KEGG classification. To further explore pathway enrichment, a KEGG enrichment bubble plot was generated (Figure 4D). The pathways with the most significantly enriched differential metabolites included “Biosynthesis of unsaturated fatty acids” and “Glycerophospholipid metabolism.” Other significantly enriched pathways involved in carbon and amino acid metabolism were “Alanine, aspartate and glutamate metabolism,” “Choline metabolism in cancer,” “Central carbon metabolism in cancer,” “Arginine biosynthesis,” “Retrograde endocannabinoid signaling,” and “Carbon metabolism.” Subsequent topological analysis of KEGG pathways (Figure 4E) identified “Biosynthesis of unsaturated fatty acids” and “Alanine, aspartate and glutamate metabolism” as the most influential and enriched pathways, suggesting that these represent the key routes through which live and inactivated FRT4 exert their metabolic regulatory effects.

**Figure 5:**
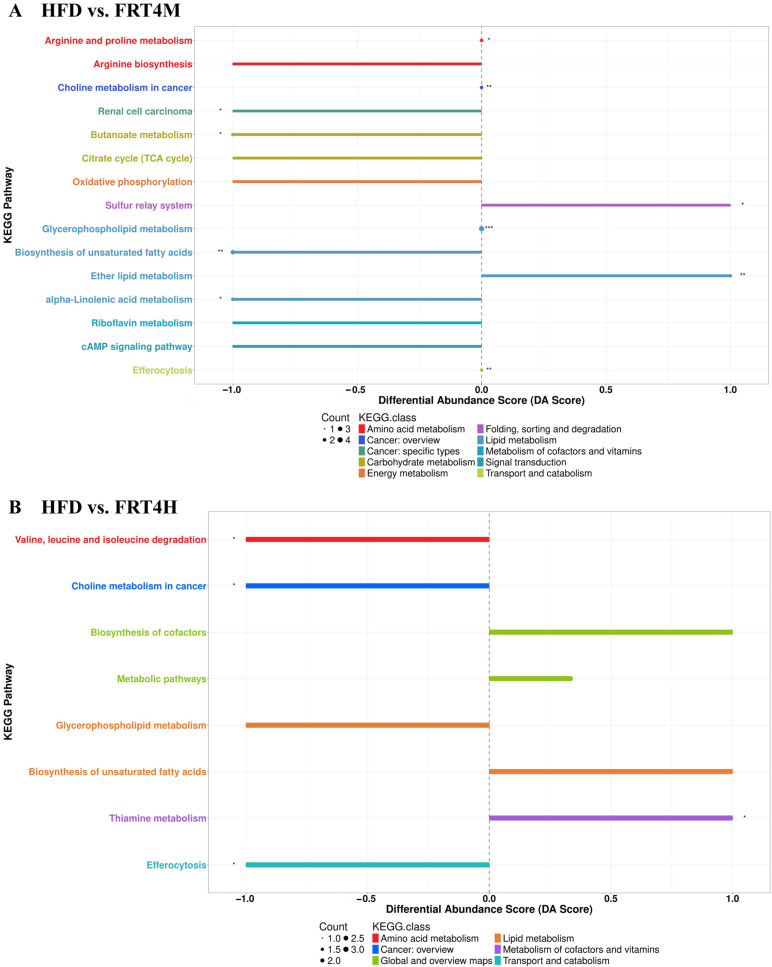
DA Score plots of KEGG pathway. (**A**) HFD vs. FRT4M. (**B**) HFD vs. FRT4H. DA Score reflects the net regulation trend of each metabolic pathway, calculated as the normalized difference between the number of upregulated and downregulated metabolites. Positive DA Scores (bars on the right) indicate net activation or upregulation of the pathway. Negative DA Scores (bars on the left) indicate net suppression or downregulation of the pathway. The symbols *, **, and *** indicate significant differences at the *p* < 0.05, *p* < 0.01, and *p* < 0.001 levels.

To validate this hypothesis, Differential Abundance Score (DA Score) analysis was performed to assess the overall metabolic pathway changes in response to live and inactivated FRT4 intervention in HFD-fed mice. This score was defined as the normalized difference between the number of significantly upregulated and downregulated metabolites in a given pathway, relative to the total number of identified metabolites in that pathway. As shown in Figure 5A, inactivated FRT4 significantly downregulated pathways such as “Biosynthesis of unsaturated fatty acids,” “Arginine biosynthesis,” and the “Citrate cycle (TCA cycle),” with the most notable suppression observed in the “Biosynthesis of unsaturated fatty acids” pathway. This indicates that this pathway is a primary mechanism by which inactivated FRT4 alleviates hepatic metabolic disturbances and obesity. In contrast, live FRT4 mainly downregulated pathways such as “Choline metabolism in cancer” and “Glycerophospholipid metabolism.” Comparative DA Score plots further confirmed that inactivated FRT4 had a greater impact on hepatic metabolism than live FRT4. In summary, HFD significantly disrupted hepatic metabolism in mice. Both live and inactivated FRT4 attenuated this disruption by modulating lipid, amino acid, and carbon metabolism pathways. Among them, inactivated FRT4 primarily exerted its metabolic regulatory effect through the “Biosynthesis of unsaturated fatty acids” pathway.

### 3.7. Inactivated Lp. plantarum FRT4 Modulates Hepatic Gene Transcription

**Figure 6:**
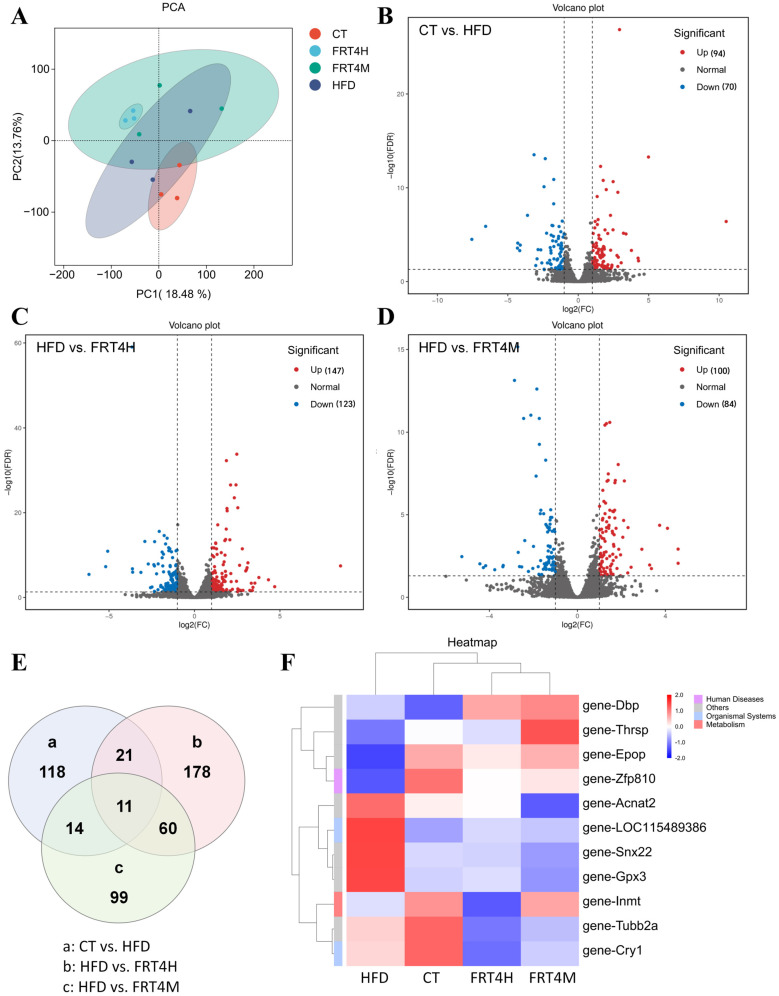
Inactivated *Lp. plantarum* FRT4 alleviates hepatic transcriptional dysregulation in HFD-fed mice. (**A**) PCA plot of liver transcriptomic profiles. (**B**) Volcano plot of DEGs between CT and HFD groups. (**C**) Volcano plot of DEGs between HFD and FRT4H groups. (**D**) Volcano plot of DEGs between HFD and FRT4M groups. (**E**) Venn diagram showing shared and unique DEGs among comparison groups. (**F**) Hierarchical clustering heatmap of DEGs (FDR < 0.05, |log_2_FC| ≥ 1). *N* = 3 mice per group.

To explore the potential mechanisms by which live and inactivated FRT4 exert their effects in HFD-fed mice, non-targeted transcriptomic analysis was performed on liver tissues. As shown in the PCA plot (Figure 6A), the HFD group exhibited a distinct transcriptional profile compared to the CT group, indicating that HFD significantly altered hepatic gene expression. Following intervention with live or inactivated FRT4, the transcriptional profiles diverged from that of the HFD group, suggesting that both treatments modulate hepatic gene expression patterns. The transcriptome of the inactivated FRT4 is more similar to that of the CT group. Volcano plots were used to visualize differentially expressed genes (DEGs) between groups (*p*-adjusted < 0.05, |log_2_FC| ≥ 1). Compared to the CT group, the HFD group had 94 significantly upregulated and 70 significantly downregulated genes (Figure 6B). In the live FRT4 group, 147 genes were upregulated and 123 were downregulated relative to the HFD group (Figure 6C). In the inactivated FRT4 group, 100 genes were upregulated and 84 were downregulated (Figure 6D). Venn diagram analysis revealed 11 DEGs commonly shared among the three comparison groups (CT vs. HFD, HFD vs. FRT4H, and HFD vs. FRT4M) (Figure 6E). Additionally, 118, 178, and 99 unique DEGs were identified in CT vs. HFD, HFD vs. FRT4H, and HFD vs. FRT4M, respectively. Hierarchical clustering of DEGs (FDR < 0.05, |log_2_FC| ≥ 1, Figure 6F) showed that the CT, LP4H, and LP4M groups clustered together in the upper dendrogram, indicating higher transcriptomic similarity among these groups. In contrast, the HFD group formed a separate cluster. These results indicate that both live and inactivated *Lp. plantarum* FRT4 can partially restore hepatic gene expression patterns disrupted by a high-fat diet, shifting them toward a healthier transcriptional profile.

**Figure 7:**
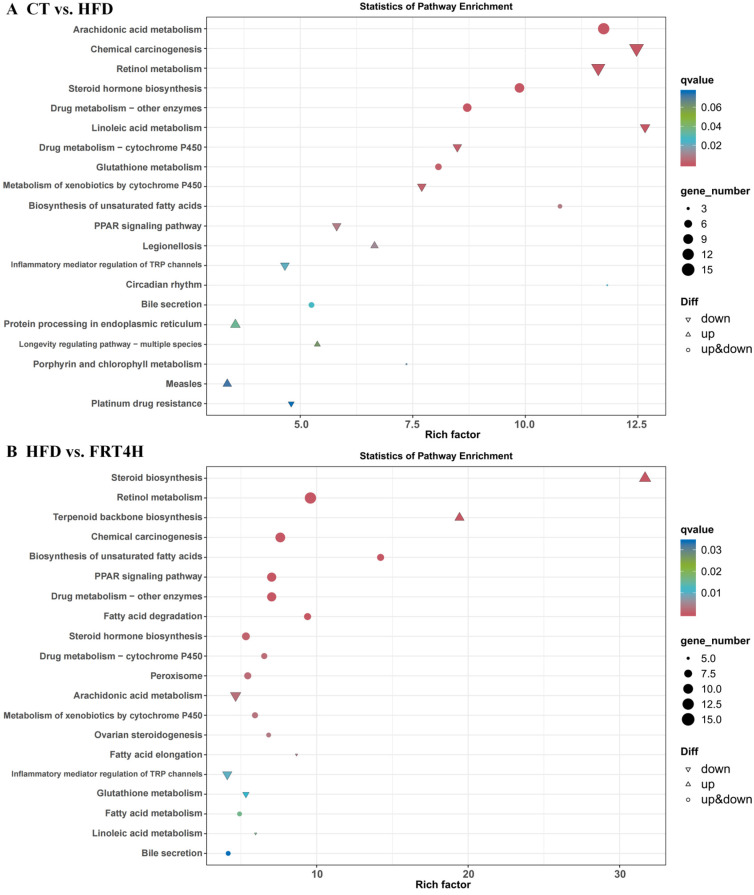

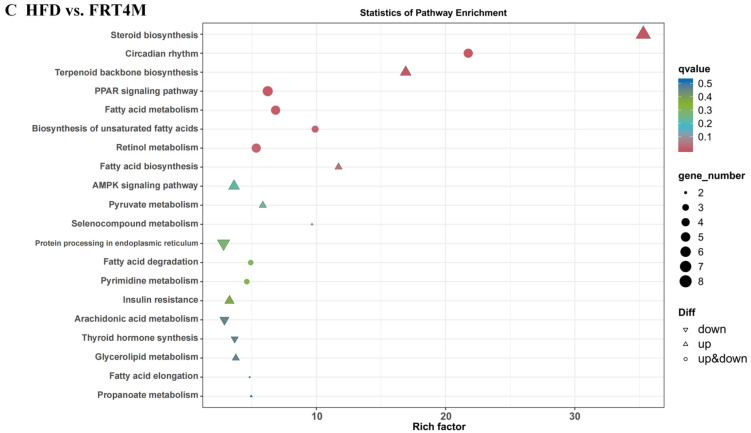
KEGG pathway enrichment analysis of differentially expressed hepatic genes among groups. (**A**) KEGG enrichment bubble plot for DEGs between CT and HFD groups. (**B**) KEGG enrichment bubble plot for DEGs between HFD and FRT4H groups. (**C**) KEGG enrichment bubble plot for DEGs between HFD and FRT4M groups. *N* = 3 mice per group.

To further investigate the functional characteristics of live and inactivated FRT4 in alleviating HFD-induced obesity, KEGG pathway enrichment analysis was performed on the differentially expressed genes (DEGs). The DEGs between the CT and HFD groups were primarily enriched in pathways including “Arachidonic acid metabolism,” “Retinol metabolism,” “Steroid hormone biosynthesis,” “Linoleic acid metabolism,” “Glutathione metabolism,” “Biosynthesis of unsaturated fatty acids,” and the “PPAR signaling pathway” (Figure 7A), indicating that HFD significantly altered hepatic metabolic pathways. Compared with the HFD group, DEGs in the live FRT4 group were mainly enriched in “Steroid biosynthesis,” “Retinol metabolism,” “Biosynthesis of unsaturated fatty acids,” “PPAR signaling pathway,” “Arachidonic acid metabolism,” “Fatty acid degradation,” “Fatty acid elongation,” “Fatty acid metabolism,” and “Steroid hormone biosynthesis” (Figure 7B). Similarly, DEGs between the HFD and inactivated FRT4 groups were enriched in “Steroid biosynthesis,” “PPAR signaling pathway,” “Fatty acid metabolism,” “Biosynthesis of unsaturated fatty acids,” “Retinol metabolism,” “Fatty acid biosynthesis,” “Fatty acid elongation,” “Pyruvate metabolism,” “Arachidonic acid metabolism,” and “Fatty acid degradation” (Figure 7C), suggesting that inactivated FRT4 modulates multiple pathways involved in fatty acid synthesis and metabolism. These results collectively demonstrate that both live and inactivated FRT4 primarily influence fatty acid synthesis and metabolism-related pathways, highlighting their regulatory roles in lipid metabolism in obese mice.

## 4. Discussion

In our previous studies, we demonstrated that live *Lp. plantarum* FRT4 effectively alleviated HFD-induced obesity in mice, including reduced body weight gain, improved lipid metabolism disorders, mitigated hepatic steatosis, and significant modulation of gut microbiota composition and function [26,32]. However, the practical application of live probiotics is often limited by issues such as instability of viability and challenges in transportation and storage. Based on our earlier findings, the present study focused on investigating the anti-obesity effects and underlying mechanisms of heat-inactivated *Lp. plantarum* FRT4, aiming to explore its potential as a postbiotic. This study systematically evaluated the multi-level roles of inactivated FRT4 in alleviating HFD-induced obesity in mice. The results demonstrated that inactivated FRT4 effectively attenuated body weight gain, reduced organ indices, improved hepatic steatosis, and significantly ameliorated metabolic phenotypes without affecting food intake. Notably, although HFD-fed mice displayed reduced food intake compared to controls, they still developed significant obesity and metabolic disturbances. This seemingly paradoxical phenomenon has been well documented and is primarily attributed to the much higher energy density of high-fat diets compared to standard chow; even with a lower food intake by weight, total caloric intake remains higher, leading to increased body weight and fat accumulation [41,42,43]. Furthermore, chronic exposure to high-fat diets can disrupt central appetite regulation through dysregulation of satiety hormones (such as leptin and insulin), as well as hypothalamic inflammation, both of which impair normal appetite and energy homeostasis [44,45]. Compared to the live FRT4 group, inactivated FRT4 exhibited comparable and even superior efficacy in some aspects. These findings suggest that inactivated FRT4 possesses independent and potent physiological regulatory effects. Collectively, this study provides new insights into the mechanisms by which inactivated probiotics exert anti-obesity effects and supports the potential application of inactivated FRT4 as a functional postbiotic in obesity intervention.

At the mechanistic level, inactivated FRT4 exhibited a pronounced modulatory effect on the gut microbial ecosystem. 16S rRNA sequencing revealed that HFD markedly disrupted the diversity and compositional structure of the gut microbiota, as evidenced by increased α-diversity indices, an elevated F/B ratio, and a decline in the abundance of several beneficial genera. Although most studies report a decrease in gut microbial α-diversity in obesity and HFD models, some have observed paradoxical increases or no significant changes [46,47]. The biological significance of increased α-diversity in HFD models remains unclear and may be influenced by factors such as animal strain, diet composition, and sampling protocols. And it may result from the expansion of non-beneficial or opportunistic taxa, reflecting a shift toward a more disordered and imbalanced community, rather than a healthy microbial ecosystem. In our study, the increased α-diversity observed in the HFD group coincided with an elevated F/B ratio and a reduction in beneficial genera, which is generally considered a sign of gut dysbiosis. Nevertheless, further mechanistic studies are needed to clarify the implications of these findings.

Intervention with inactivated FRT4 effectively reversed these alterations by reducing the F/B ratio, increasing the relative abundance of beneficial microbes such as *Lactobacillus*, *Bacteroides*, *Alistipes*, and *Muribaculaceae*, while suppressing potentially harmful taxa including *unclassified_Lachnospiraceae*, *Coriobacteriaceae*, and *Bilophila*. These changes contributed to the restoration of gut microbial homeostasis. The microbial shifts may partly explain the enhanced SCFA levels observed in the FRT4M group, as several of the enriched genera (e.g., *Bacteroides*, *Alistipes*, and *Muribaculaceae*) are established SCFA producers, which have been associated with anti-inflammatory and disease-alleviating effects [48,49,50,51]. This indicates that the increased SCFA production is closely linked to the specific microbial changes induced by heat-inactivated FRT4. Interestingly, we observed that the relative abundance of the genus *Lactobacillus* decreased significantly in the HFD group compared to the CT group, consistent with previous reports showing that HFD can disrupt gut microbial homeostasis and reduce beneficial taxa, including *Lactobacillus* [52]. Interestingly, the abundance of *Lactobacillus* was markedly restored in the group receiving heat-inactivated FRT4, and to a lesser extent in the group supplemented with live FRT4. This differential response suggests that the beneficial effects of FRT4 on gut microbiota composition may not solely depend on bacterial viability. Postbiotics have been shown to retain immunomodulatory and barrier-protective functions through structural components such as peptidoglycans and surface proteins [28,53,54], which may promote the growth of endogenous *Lactobacillus* species. In contrast, *unclassified_Lachnospiraceae*, *Coriobacteriaceae*, and *Bilophila* are considered potential pathobionts, closely linked to obesity, inflammation, and even tumorigenesis [55,56,57,58,59,60]. Importantly, inactivated FRT4 outperformed the live strain in modulating microbial structure at both the F/B ratio and genus levels, suggesting that specific bacterial components may influence host–microbiota interactions.

The improved microbial profile further facilitated the synthesis and accumulation of SCFAs. We observed that inactivated FRT4 significantly increased the levels of several SCFAs, particularly acetic acid, isobutyric acid, and hexanoic acid. As key microbial metabolites, SCFAs not only serve as crucial energy sources for intestinal epithelial cells but also participate in fat metabolism, glucose homeostasis, and inflammation regulation by activating receptors such as GPR41 and GPR43 [61,62,63,64]. Importantly, these SCFAs can enter the portal circulation and reach the liver, where they influence hepatic metabolic programming [65,66]. Therefore, the SCFA-promoting effects of inactivated FRT4 provide compelling metabolic evidence for its anti-obesity activity and further support the classical regulatory axis of “microbiota–metabolites–host metabolism”.

To further investigate the downstream metabolic consequences of FRT4-mediated microbiota remodeling and SCFA production, we conducted untargeted liver metabolomics. The analysis revealed substantial improvements in hepatic lipid, amino acid, and carbon metabolic pathways, indicating that SCFAs may act as critical molecular mediators linking gut microbial activity to hepatic metabolic reprogramming. These findings underscore the central role of the gut–liver axis in maintaining systemic metabolic homeostasis during dietary interventions. As previously reported, HFD and obesity are known to induce hepatic metabolic disorders [67]. KEGG enrichment analysis of differential metabolites revealed that HFD disrupted liver metabolism, particularly lipid-related pathways such as unsaturated fatty acid biosynthesis and glycerophospholipid metabolism. Supplementation with both live FRT4 and inactivated FRT4 partially reversed these HFD-induced metabolic disturbances, with the effects of inactivated FRT4 being more pronounced, especially in significantly downregulating the “Biosynthesis of unsaturated fatty acids” pathway. This suggests that inactivated FRT4 may alleviate hepatic lipid accumulation and inflammatory responses by suppressing endogenous lipid synthesis.

Although unsaturated fatty acids (UFAs), particularly polyunsaturated fatty acids (PUFAs), are generally regarded as metabolically protective due to their anti-inflammatory properties and ability to improve insulin sensitivity [68,69], the endogenous biosynthesis of monounsaturated fatty acids (MUFAs), especially mediated by stearoyl-CoA desaturase 1 (SCD1), plays a pivotal role in hepatic lipogenesis [70]. SCD1 is a key rate-limiting enzyme that catalyzes the desaturation of saturated fatty acids such as palmitic acid (C16:0) and stearic acid (C18:0) into MUFAs like palmitoleic acid (C16:1) and oleic acid (C18:1), which are major components of triglyceride synthesis. Upregulation of SCD1 and MUFAs has been consistently linked to increased lipid storage, hepatic steatosis, and insulin resistance in obese models [70,71,72]. In our study, the observed downregulation of the “biosynthesis of unsaturated fatty acids” pathway at both the metabolomic and transcriptomic levels likely reflects a suppression of SCD1-mediated desaturation activity. This may represent a beneficial metabolic adaptation aimed at reducing de novo triglyceride formation and lipid deposition in hepatocytes. Previous studies have shown that genetic deletion or pharmacological inhibition of SCD1 protects against diet-induced obesity and metabolic disorders [73,74]. Therefore, the suppression of this pathway by inactivated FRT4 may reduce hepatic lipotoxicity by limiting MUFA synthesis and shifting lipid metabolism toward oxidation rather than storage. Therefore, inhibition of this pathway by inactivated FRT4 may alleviate hepatic lipid overload and inflammation. This metabolic reprogramming is likely mediated by host–microbiota interactions, including SCFA-induced signaling and modulation of innate immune pathways [71]. Moreover, inactivated FRT4 significantly affected amino acid metabolism and carbon utilization pathways, such as arginine biosynthesis and the TCA cycle, suggesting a broader regulatory role in hepatic energy metabolism beyond lipid handling.

Notably, live and inactivated FRT4 exhibited distinct effects on metabolic pathways, indicating that they may act through different mechanisms. FRT4H mainly influenced glycerophospholipid metabolism and choline metabolism in cancer, which may be associated with immune modulation, membrane remodeling, and insulin sensitivity [75,76]. In contrast, inactivated FRT4 exerted broader systemic effects across pathways involved in lipid synthesis, amino acid biosynthesis, and the TCA cycle. These findings support the emerging concept of postbiotics as effective metabolic modulators [77]. The superior metabolic regulatory effects of inactivated FRT4 over its live form highlight its potential as a promising candidate for the prevention and treatment of metabolic disorders.

Transcriptomic analysis further elucidated the molecular mechanisms underlying the regulatory effects of inactivated FRT4. PCA and hierarchical clustering heatmaps revealed that HFD markedly altered the hepatic gene expression profile. However, interventions with both live and inactivated FRT4 shifted the transcriptomic patterns away from the HFD group and closer to those of the control group, suggesting a restorative effect of FRT4 on hepatic transcriptional homeostasis. KEGG enrichment analysis showed that HFD significantly affected multiple signaling pathways associated with lipid metabolism and oxidative stress, including “Arachidonic acid metabolism”, “Retinol metabolism”, “Steroid hormone biosynthesis”, “Linoleic acid metabolism”, “Glutathione metabolism”, and “PPAR signaling pathway”. These pathways are closely related to lipid accumulation, inflammatory responses, and oxidative stress in obesity [78,79,80,81,82]. Following FRT4 intervention (particularly in the inactivated FRT4 group), differentially expressed genes were significantly enriched in pathways associated with fatty acid synthesis and degradation, such as “Biosynthesis of unsaturated fatty acids”, “Fatty acid metabolism”, “Fatty acid biosynthesis”, “Fatty acid elongation”, “Pyruvate metabolism”, “Fatty acid degradation”, and “PPAR signaling pathway”. These transcriptomic changes indicate that both live and inactivated FRT4 play key roles in modulating the dynamic balance between lipid synthesis and breakdown, thereby contributing to the improvement of hepatic lipid homeostasis. Notably, the “Biosynthesis of unsaturated fatty acids” pathway was also significantly enriched in the metabolomic analysis, further supporting its central role in mediating the protective effects of inactivated FRT4 against HFD-induced metabolic disturbances. Moreover, the “PPAR signaling pathway”, a major regulatory hub for lipid synthesis, transport, and insulin sensitivity, may represent a critical mechanism by which FRT4 improves lipid metabolism and alleviates hepatic steatosis [83,84,85]. Inactivated FRT4 also influenced pyruvate metabolism and other carbon source utilization pathways, implying that it may further mitigate HFD-induced metabolic abnormalities by modulating energy metabolism and substrate flux.

Compared with other lactic acid bacteria (LAB) strains that are phylogenetically related to FRT4, such as *Lactobacillus brevis*, *Lacticaseibacillus rhamnosus*, and *Limosilactobacillus reuteri*, the inactivated FRT4 exhibits both shared and unique features in its anti-obesity effects. Several LAB strains, in both live and heat-inactivated forms, have been shown to alleviate obesity-related metabolic disturbances via gut microbiota modulation, SCFA production, and regulation of host signaling pathways. For instance, heat-killed *Lactobacillus brevis* KB290 has been shown to suppress diet-induced visceral fat accumulation and improve metabolic abnormalities and gut microbiota alterations caused by a high-fat diet [86]. Live *Lactobacillus rhamnosus* GG alleviated diet-induced non-alcoholic fatty liver disease (NAFLD) in rats by reducing oxidative stress and inflammation [87]. Similarly, heat-inactivated *Lactobacillus reuteri* GMNL-263 exhibited comparable effects to its live counterpart in alleviating obesity-related metabolic dysfunctions by reducing insulin resistance and improving hepatic steatosis [88]. These findings are consistent with our study, wherein inactivated *Lp. plantarum* FRT4 mitigated lipid accumulation and obesity in mice through modulation of gut microbiota and hepatic lipid metabolism. However, our multi-omics analysis revealed that FRT4M exerted a particularly strong regulatory effect on the “biosynthesis of unsaturated fatty acids” pathway, both at the metabolomic and transcriptomic levels, which was a distinctive feature not commonly reported for other LAB strains. This suggests that *Lp. plantarum* FRT4, especially in its inactivated form, may possess strain-specific metabolic reprogramming potential and unique postbiotic properties within the LAB family. These differences may be attributed to genomic diversity, cell wall structure, or host–microbe interaction profiles. Together, these findings highlight the importance of strain-level evaluation in probiotic and postbiotic research.

Overall, inactivated FRT4 exerts anti-obesity effects through a multi-layered, synergistic mechanism. Its primary actions include modulating gut microbiota composition and restoring microbial homeostasis; promoting the production of SCFAs, thereby regulating host energy metabolism and lipid balance; remodeling hepatic metabolic networks to correct abnormalities in lipid, carbohydrate, and amino acid metabolism; and regulating the expression of key genes involved in lipid metabolism and inflammation-related signaling pathways. These mechanisms act in concert to establish a systemic framework through which inactivated FRT4 alleviates obesity and associated metabolic disorders. Although inactivated FRT4 lacks viability, its bioactivity is likely mediated by structural and molecular components retained after heat treatment, such as peptidoglycans and short peptides [54,89]. These components can interact with host receptors or modulate immune and metabolic pathways, contributing to the postbiotic-like effects observed [28,53,54]. Notably, integrative analysis of the transcriptomic and metabolomic datasets revealed that the “biosynthesis of unsaturated fatty acids” pathway was consistently and significantly downregulated in the FRT4M intervention group at both gene expression and metabolite levels. This convergence highlights a coordinated suppression of hepatic de novo lipogenesis. The alignment of transcriptomic and metabolomic alterations reinforces the conclusion that inactivated FRT4 exerts its anti-obesity effects in part through multi-layered regulation of hepatic lipid metabolic pathways.

Despite the comprehensive multi-omics insights provided in this study, several limitations remain. First, the findings are based on a mouse model, and the translational relevance to humans requires further clinical validation. Nevertheless, heat-inactivated probiotics such as FRT4 offer unique advantages (including enhanced safety, stability, and ease of formulation) that make them promising candidates for human use. The observed gut–liver axis modulation and metabolic improvements highlight potential therapeutic mechanisms that warrant investigation in future clinical trials. Second, the bioactive components of inactivated FRT4 have yet to be identified. Future studies incorporating proteomics and lipidomics approaches are warranted to elucidate its key functional constituents and molecular targets. Third, serum insulin levels and insulin resistance indices (e.g., HOMA-IR) were not directly measured. Although multi-omics analyses indicated improvements in glucose and lipid metabolism, direct assessment would provide a more complete understanding of the underlying mechanisms. Moreover, while the 16-week intervention is standard in murine models, it may not reflect long-term efficacy or safety, and extended studies are warranted. Finally, only a single dosage was tested; future work should explore dose–response relationships to optimize therapeutic potential.

## 5. Conclusions

In this study, we systematically evaluated the anti-obesity effects of both live and heat-inactivated *Lp. plantarum* FRT4 in an HFD-induced mouse model of obesity. The results demonstrated that both forms of FRT4 effectively alleviated obesity and associated metabolic disturbances, with the inactivated strain exhibiting comparable or even superior efficacy across multiple parameters. Inactivated FRT4 intervention significantly suppressed body weight gain, reduced organ hypertrophy, and improved lipid metabolism in both serum and liver. Additionally, it restored gut microbiota composition and promoted the production of SCFAs. Multi-omics analyses revealed that inactivated FRT4 exerted its anti-obesity effects by regulating hepatic lipid biosynthesis, amino acid metabolism, and the expression of related genes, particularly through the downregulation of key metabolic pathways such as “Biosynthesis of unsaturated fatty acids”. These findings suggest that FRT4M may orchestrate a systemic regulation of energy metabolism via the gut–liver axis. Collectively, this study highlights the potential of inactivated FRT4 as a novel postbiotic with promising applications in functional foods and the prevention or treatment of metabolic disorders.

## Data Availability

The original contributions presented in the study are included in the article, and further inquiries can be directed to the corresponding authors. Raw data of 16S rRNA gene sequencing were deposited in the NGDC GSA database (accession number: CRA027577). Raw data of liver transcriptome sequencing were deposited in the NGDC GSA database (accession number: CRA027617).
